# Persistent Organic Pollutants and Plasma Markers of Neurodegeneration in SOL-INCA and HCHS/SOL

**DOI:** 10.1093/geroni/igaf122.579

**Published:** 2025-12-31

**Authors:** Julia Bauer, Mary Turyk, Victoria Persky, Charles DeCarli, Bharat Thyagarajan, Wassim Tarraf, Hector Gonzalez

**Affiliations:** University of Illinois Chicago, Chicago, Illinois, United States; School of Public Health, University of Illinois at Chicago, Chicago, Illinois, United States; School of Public Health, University of Illinois at Chicago, Chicago, Illinois, United States; University of California, Davis, Sacramento, California, United States; University of Minnesota, Minneapolis, Minnesota, United States; Wayne State University, Detroit, Michigan, United States; University of California, San Diego, San Diego, California, United States

## Abstract

There is an urgent need to identify modifiable risk factors during early stages of neurodegeneration to reduce dementia risks. Persistent organic pollutants (POPs), including DDT and other organochlorine pesticides (OCPs) and polychlorinated biphenyls (PCBs), are environmental contaminants that accumulate in human tissues and have neurotoxic potential and sex-specific effects. We examined sex-specific associations between POPs and plasma biomarkers of neurodegeneration. This study included 1,837 participants (mean age 55 years) from the Hispanic Community Health Study/Study of Latinos with serum POPs measurements at baseline (2008–2011; V1) and a 7-year follow-up (2015–2018; V2). Serum POPs (DDT, OCPs, PCBs) were quantified using gas chromatography-isotope dilution high-resolution mass spectrometry. At V2, plasma biomarkers of neurodegeneration were measured at the University of Minnesota, including AD-related proteins (amyloid beta, phosphorylated tau 181) and non-specific markers of neurodegeneration (Glial Fibrillary Acid Protein (GFAP), neurofilament light chain (NfL)). Covariate-adjusted associations between lipid-adjusted POPs and neurodegeneration biomarkers were estimated using linear regression with survey weights. Sex differences were explored using sex stratified models and sex-POP interaction p-values in main models. Sex-specific associations were observed between POPs and non-specific neurodegeneration markers. Among females, DDT was associated with higher GFAP concentrations (2nd vs. 1st tertile: beta=0.29 (95% CI: 0.07, 0.51); 3rd vs. 1st tertile: beta=0.58 (0.25, 0.91); p-values for sex*DDT interaction <0.01). Among males, OCP was associated with increased NfL (2nd vs. 1st tertile: beta=0.33 (0.17, 0.49)); p-interaction sex*OCP <0.001). Findings suggest sex differences in the association between POPs and biomarkers of neurodegeneration.

